# Matrix Metalloproteinase-9 Relationship With Infarct Growth and Hemorrhagic Transformation in the Era of Thrombectomy

**DOI:** 10.3389/fneur.2020.00473

**Published:** 2020-06-09

**Authors:** Laura Mechtouff, Thomas Bochaton, Alexandre Paccalet, Claire Crola Da Silva, Marielle Buisson, Camille Amaz, Morgane Bouin, Laurent Derex, Elodie Ong, Yves Berthezene, Omer Faruk Eker, Nathalie Dufay, Nathan Mewton, Michel Ovize, Norbert Nighoghossian, Tae-Hee Cho

**Affiliations:** ^1^Stroke Department, Pierre Wertheimer Hospital, Hospices Civils de Lyon, Bron, France; ^2^CarMeN, INSERM U.1060/Université Lyon1/INRA U. 1397/INSA Lyon/Hospices Civils Lyon, Université de Lyon, Lyon, France; ^3^Cardiac Intensive Care Unit, Louis Pradel Hospital, Hospices Civils de Lyon, Bron, France; ^4^Clinical Investigation Center, INSERM 1407, Louis Pradel Hospital, Hospices Civils de Lyon, Bron, France; ^5^Cellule Recherche Imagerie, Louis Pradel Hospital, Hospices Civils de Lyon, Bron, France; ^6^Neuroradiology Department, Pierre Wertheimer Hospital, Hospices Civils de Lyon, Bron, France; ^7^CREATIS, CNRS UMR 5220, INSERM U1044, University Lyon 1, Lyon, France; ^8^NeuroBioTec, CRB, Pierre Wertheimer Hospital, Hospices Civils de Lyon, Bron, France

**Keywords:** stroke, matrix metalloproteinase 9, MRI, thrombectomy, thrombolytic therapy

## Abstract

**Objective:** To assess the relationship between matrix metalloproteinase 9 (MMP-9), a proteolytic enzyme involved in the breakdown of the blood-brain barrier, and infarct growth and hemorrhagic transformation in acute ischemic stroke (AIS) with large vessel occlusion (LVO) in the era of mechanical thrombectomy (MT) using the kinetics of MMP-9 and sequential magnetic resonance imaging (MRI).

**Methods:** HIBISCUS-STROKE is a cohort study including AIS patients with LVO treated with MT following admission MRI. Patients underwent sequential assessment of MMP-9, follow-up CT at day 1, and MRI at day 6. The CT scan at day 1 classified any hemorrhagic transformation according to the European Co-operative Acute Stroke Study-II (ECASS II) classification. Infarct growth was defined as the difference between final Fluid-Attenuated Inversion Recovery volume and baseline diffusion-weighted imaging volume. Conditional logistic regression analyses were adjusted for main confounding variables including reperfusion status.

**Results:** One hundred and forty-eight patients represent the study population. A high MMP-9 level at 6 h from admission (H6) (*p* = 0.02), a high glucose level (*p* = 0.01), a high temperature (*p* = 0.04), and lack of reperfusion (*p* = 0.02) were associated with infarct growth. A high MMP-9 level at H6 (*p* = 0.03), a high glucose level (*p* = 0.03) and a long delay from symptom onset to groin puncture (*p* = 0.01) were associated with hemorrhagic transformation.

**Conclusions:** In this MT cohort study, MMP-9 level at H6 predicts infarct growth and hemorrhagic transformation.

## Introduction

Ischemia-reperfusion injury in stroke is defined as a biochemical cascade causing a deterioration of ischemic brain tissue that parallels and antagonizes the beneficial effect of reperfusion (1). A key feature of this process is the proteolytic breakdown of the blood-brain barrier (BBB) vasculature. The increase of BBB permeability is mediated by activation of matrix metalloproteinase (MMP), and especially MMP-9 (2, 3).

So far, the importance of MMP-9 on infarct growth and risk of hemorrhagic transformation has not been explored in relation to mechanical thrombectomy (MT). In the context of intravenous (IV) thrombolysis, early increase of MMP-9 expression may promote hemorrhagic transformation but also infarct growth with subsequent influence on neurological disability (4–12). Since restoration of the blood supply following MT might be more abrupt and potentially cause greater BBB damage despite a timely and successful reperfusion, an appropriate assessment of MMP-9 activity in this setting may provide additional insight into reperfusion injury related to MT (13). We sought to determine whether early MMP-9 level is associated with infarct growth and hemorrhagic transformation. For this purpose, a sequential assessment of MMP-9 and ischemic damage using MRI was implemented in the setting of MT.

## Methods

### Study Population

**Figure 1:**
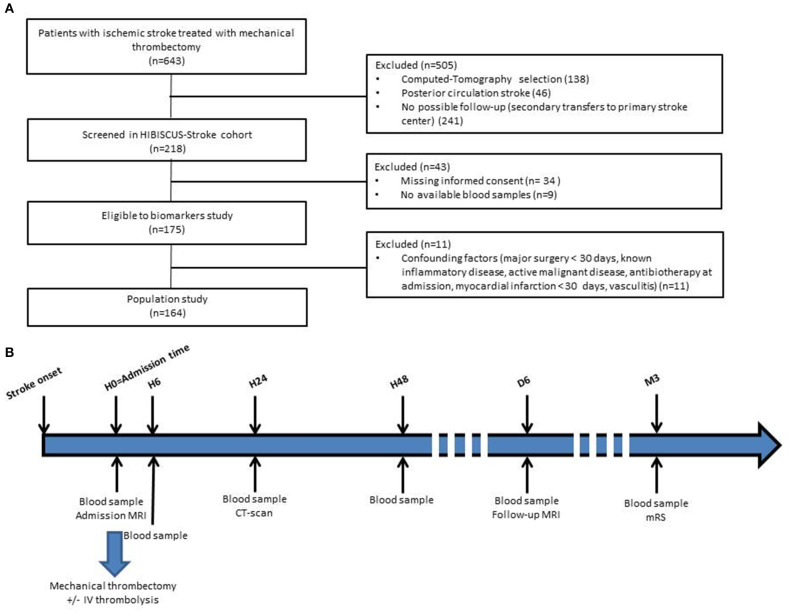
Flow-chart of patient selection **(A)** and timeline of HIBISCUS-STROKE cohort from admission **(B)** (H0, admission; H6, hour 6; H24, hour 24; H48, hour 48; M3, month 3; IV, intravenous; MRI, magnetic resonance imaging; CT, computed tomography; mRS, modified Rankin Scale).

HIBISCUS-STROKE is an ongoing cohort study including all patients admitted since October 2016 in the Lyon Stroke Department for an acute ischemic stroke (AIS) with large vessel occlusion (LVO) treated either within 0–6 h or 6–24 h time window with MT following brain magnetic resonance imaging (MRI) assessment. Patients with Computed-Tomography at admission, with posterior circulation stroke, without follow-up planned in our institution (secondary transfers to primary stroke center), without informed consent and without available blood samples were excluded from the HIBISCUS-STROKE cohort. Among patients included in the HIBISCUS-STROKE cohort, those with known inflammatory disease, active malignancy, vasculitis, antibiotics at admission, myocardial infarction, or major surgery in the 30 previous days were excluded in order not to skew the results of the biomarkers analysis. All patients underwent a sequential assessment of systemic MMP-9 level. Peripheral blood samples were collected from each patient: at admission (H0), 6 h (H6), 24 h (H24), and 48 h (H48) from admission. A CT scan was performed at day 1 in order to rule out any hemorrhagic transformation. Final infarct size was assessed on follow-up MRI at day 6 (Figure 1).

Baseline data on demographic characteristics, lifestyle risk factors, medical history, and use of medications were collected at hospital admission. Neurological status was assessed by board certified neurologists using National Institute of Health Stroke Scale (NIHSS) score at admission, and the modified rankin scale (mRS) score at 3 months during a face-to-face follow-up visit. Poor outcome was defined as 3-month mRS score > 2. Stroke subtype was classified according to the Trial of Org 10,172 in Acute Stroke Treatment (TOAST) criteria (14).

The study was approved by the local ethics committee and all subjects or their relatives signed an informed consent form.

### Neuroimaging

All MRIs were performed with 1.5-Tesla Intera or 3-Tesla Achieva scanners (Philips, Best, Netherlands). The admission MRI protocol included fluid-attenuated inversion recovery (FLAIR), T2-gradient echo, diffusion-weighted imaging (DWI), time-of-flight MRA, and perfusion-weighted imaging. The CT scan at day 1 classified any hemorrhagic transformation according to the European Co-operative Acute Stroke Study-II (ECASS II) classification (15). The follow-up MRI protocol at day 6 included FLAIR sequence. A stroke neurologist (T-H. C.) with expertise in neuroradiology, blinded to clinical and laboratory data, independently reviewed both admission and follow-up MRI using a dedicated post-processing work station (3D slicer software). The acute ischemic lesion was segmented from the baseline DWI with a semi-automated method (3D Slicer: https://www.slicer.org/) by using both a validated ADC threshold (ADC <620 × 10^−6^ mm^2^/s) and visual assessment of b1000 images. The final infarct was identified on day-6 FLAIR images using 3D Slicer. Lesion volumes were subsequently calculated from the segmentation masks. Infarct growth was defined as the difference between final volume on the FLAIR-sequence and baseline volume on the DWI-sequence. Alberta Stroke Program Early CT score (ASPECTS) was measured on baseline DWI (16). Successful reperfusion was defined as thrombolysis in cerebral infarction score (TICI) ≥2b (17).

### Blood Sampling Protocol

White blood cells (WBC) count and high sensitivity C-reactive protein (hsCRP) were routinely measured at admission. MMP-9 level was measured using DuoSet® ELISA Development Kits (R&D Systems). Sera were prepared and stored at −80°C within a 3 h delay at the NeuroBioTec biobank (CRB-HCL: BB-0033-00046, France). All samples were thawed only once for study measurements. Serum samples were diluted at 1/2,000. Sensitivity was 12.2 pg/mL.

### Statistical Analysis

Continuous variables are expressed as means (standard deviation [SD]) or medians (interquartile range [IQR]), and categorical variables as percentages. Medians were compared using the Mann–Whitney or Kruskall–Wallis test for independent samples. The Wilcoxon signed rank test was performed for matched samples. Percentages were compared using the Fishers exact test. Spearman correlation coefficients (*r*) were calculated between variables. Analyses were focused on the early MMP-9 peak at H6. Normality of distributions was assessed graphically and with the Shapiro–Wilk test. As MMP-9, infarct growth, WBC count, and hsCRP were not normally distributed, we dichotomized them according to their median. The association between MMP-9 level at H6 and infarct growth and hemorrhagic transformation was measured by calculating crude odds ratios (ORs) and 95% CIs using conditional logistic regression analyses. A multiple logistic regression model was performed to detect independent markers of infarct growth or any hemorrhagic transformation. Covariates with a significant univariate association with infarct growth or hemorrhagic transformation were included in each multivariate model along with other potential predictors independent of their univariate *p*-value, selected a priori. A backward selection procedure was chosen. The models were adjusted for:

– infarct growth: age, sex, glucose level, temperature, baseline volume on the DWI-sequence, stroke onset to groin puncture time, IV thrombolysis, and reperfusion status (NIHSS score and systolic blood pressure not retained by the backward selection),
– hemorrhagic transformation: sex, glucose level, stroke onset to groin puncture time, IV thrombolysis, and baseline volume on the DWI-sequence (age, NIHSS score, and systolic blood pressure not retained by the backward selection).

Two-tailed *p* < 0.05 was considered to be statistically significant. The data were analyzed with Stata Version 15™ (STATACORP, COLLEGE STATION, TEXAS 77845 USA).

### Data Availability Statement

Further anonymized data can be made available to qualified investigators on request to the corresponding author.

## Results

### HIBISCUS-STROKE Cohort

Between October 2016 to April 2019, 148 patients met the inclusion criteria (Figure 1). Baseline and follow-up MRI were available and interpretable for 127 (77.4%) patients. The main clinical and imaging characteristics are shown in Table 1. Mean age was 69 ± 15. Sixty percent of patients were men. Median NIHSS score on admission was 15 [9–19]. Median infarct growth was 3.4 cc [−1.3 to 24.6]. Hemorrhagic transformation occurred in 40 patients (27.6%). Ninety-one (61.5%) patients had a good outcome (mRS score 0–2) at 3 months. No patient was lost at the 3-month follow-up.

**Table 1:** Characteristics of the study population.

		**MMP-9 level at H6**		
	**All (*n* = 148)**	**Low-level (MMP-9 H6 ≤ 775 ng/mL) (*n* = 74)**	**High-level (MMP-9 H6 > 775 ng/mL) (*n* = 74)**	***p*-value**
Age, years	69 ± 15	70 ± 14	67 ± 16	0.15
Male, *n* (%)	89 (60.1)	46 (62.2)	43 (58.1)	0.74
Prestroke mRS score > 2	8 (5.4)	3 (4.1)	5 (6.8)	0.72
Hypertension	69 (46.6)	37 [50]	32 [43.2]	0.51
Diabetes	25 (16.9)	11 [14.9]	14 [18.9]	0.66
Hyperlipidemia	38 (25.7)	20 [27]	18 [24.3]	0.71
Current smoking	29 (19.6)	12 [16.2]	17 [23.0]	0.41
Baseline NIHSS score	15 [9–19]	13 [7–19]	16 [12–19]	0.05
SBP, mmHg	140.1 ± 22.2	141.9 ± 23.8	138.3 ± 20.6	0.39
DBP, mmHg	77.6 ± 17.4	78.0 ± 18.4	77.2 ± 16.6	0.91
Baseline temperature	36.5 ± 0.6	36.5 ± 0.5	36.5 ± 0.7	0.46
Glucose level, mmol/L	6.27 [5.61–7.59]	6.00 [5.56–6.99]	6.44 [5.78–7.92]	0.28
hsCRP at admission, mg/L	3.3 [1.6–7.8]	3 [1.3–5.9]	3.5 [1.8–11.4]	0.13
WBC count at admission, 10^9^/L	8.3 [6.6–10.0]	7 [5.9–9.0]	9.2 [8.0–11.1]	**<0.01**
Etiology				0.25
Cardioembolism	79 (53.4)	39 (52.7)	39 (52.7)	
LAA	24 (16.2)	9 (12.2)	12 (16.2)	
Other	16 (10.8)	11 (14.9)	4 (5.4)	
Undetermined	29 (19.6)	15 (20.3)	19 (25.7)	
IV thrombolysis	78 (52.7)	37 (50)	41 (55.4)	0.62
Thrombus location				
M1 MCA	118 (79.7)	57 (75.7)	62 (83.8)	0.31
segment				
M2 MCA	28 (18.9)	17 (23.0)	11 (14.9)	0.29
segment				
Intracranial ICA	44 (29.7)	21 (28.4)	23 (31.1)	0.86
Tandem occlusion	30 (20.3)	15 (20.3)	15 (20.3)	1
ASPECTS	7 [6–8]	8 [6–9]	7 [6–8]	0.13
DWI lesion volume, cc	17.4 [5.7–44.2]	13.3 [4.9–34.8]	23.0 [8.0–46.2]	0.18
Reperfusion (TICI2b-3)	123 (83.1)	63 (85.1)	60 (81.1)	0.66
Onset to admission, min	117 [70–282]	124 [73–302]	115 [70–245]	0.72
Onset to groin puncture, min	222 [155–373]	230 [156–403]	218 [155–320]	0.57
Onset to reperfusion, min	255 [195–378]	256 [195–414]	247 [195–358]	0.67
FLAIR lesion volume, cc	26.1 [7.8–61.1]	16.7 [5.1–41.4]	38.7 [13.6–85.7]	**0.01**
Infarct growth, cc	3.3 [−1.3 to 22.7]	0.8 [−4.4 to 14.8]	4.9 [0.5 to 36.2]	**0.02**
Any hemorrhagic transformation	40 (27.6)	14 (18.9)	26 (36.6)	**0.03**
PH type 1 or 2	3 (2.1)	2 (2.7)	1 (1.4)	1
SAH	4 (2.8)	1 (1.4)	3 (4.2)	0.36
mRS score 0–2	91 (61.5)	46 (62.2)	45 (60.8)	1

*MMP-9, matrix metalloproteinase 9; H6, hour 6; NIHSS, National Institute of Health Stroke Score; SBP, systolic blood pressure; DBP, diastolic blood pressure; hsCRP, High sensitivity C-reactive protein; WBC, white blood cells; LAA, Large-artery atherosclerosis; IV, intravenous; mRS, modified rankin scale; MCA, middle-cerebral-artery segment; ICA, intracranial carotid artery; ASPECTS, Alberta Stroke Program Early CT score; DWI, diffusion-weighted sequence; TICI, thrombolysis in cerebral infarction score; FLAIR, Fluid Attenuated Inversion Recovery; PH, Parenchymal hematoma; SAH, Subarachnoid hemorrhage. Variables are displayed as absolute number (percentage of column total); mean ± SD; or median (25th−75th percentiles) as appropriate. Significant values are shown in bold*.

**Figure 2:**
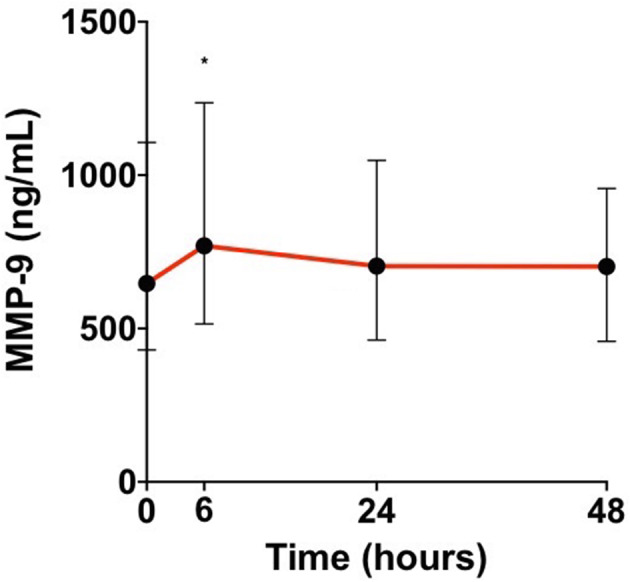
Median matrix metalloproteinase 9 (MMP-9) levels in patient's sera at admission, 6, 24, and 48 h from admission [H0, admission; H6, hour 6; H24, hour 24; H48, hour 48; Wilcoxon test for matched samples comparing MMP-9 levels at each time with the time before (*p < 0.05)].

In our population, MMP-9 levels peaked early at 6 h from admission (*p* = 0.04; Figure 2).

### MMP-9 and Infarct Growth

In univariate analyses, a high glucose level [OR = 1.25 (1.02–1.54); *p* = 0.03], a long delay from symptom onset to groin puncture [OR = 1.05 (1.00–1.11) per 30 min increase; *p* = 0.04), lack of IV thrombolysis [OR = 0.42 (0.20–0.88); *p* = 0.02] and lack of reperfusion [OR = 0.27 (0.09–0.79); *p* = 0.02] were associated with infarct growth. A high WBC count [OR = 1.30 (0.53–3.17); *p* = 0.56] and a high hsCRP level [OR = 0.69 (0.28–1.69); *p* = 0.42] at admission were not associated with infarct growth. After adjustment for main confounding variables, a high MMP-9 level at H6 [3.43 (1.23–9.55); *p* = 0.02], a high glucose level [1.43 (1.08–1.90); *p* = 0.01], a high temperature [2.55 (1.03–6.29); *p* = 0.04], and lack of reperfusion [0.16 (0.03–0.77); *p* = 0.02] were independently associated with infarct growth (Table 2).

**Table 2:** Predictors of infarct growth and any hemorrhagic transformation in univariate and multivariate analyses.

	**crude OR** **[95% CI]**	***p*-value**	**adjusted OR** **[95% CI]**	***p*-value**
**Infarct growth**				
High vs low MMP-9 level at H6	1.93 (0.92–4.01)	0.08	3.43 (1.23–9.55)	**0.02**
Glucose level^1^	1.25 (1.02–1.54)	**0.03**	1.43 (1.08–1.90)	**0.01**
Temperature^2^	1.78 (0.93–3.41)	0.08	2.55 (1.03–6.29)	**0.04**
Reperfusion (TICI 2b-3)	0.27 (0.09–0.79)	**0.02**	0.16 (0.03–0.77)	**0.02**
**Any hemorrhagic transformation**				
High vs. low MMP-9 level at H6	2.48 (1.16–5.27)	**0.02**	2.91 (1.14–7.42)	**0.03**
Glucose level^1^	1.10 (0.97–1.24)	0.14	1.20 (1.02–1.42)	**0.03**
Onset to groin puncture time^3^	1.04 (1.00–1.09)	0.06	1.08 (1.02–1.14)	**0.01**

*OR, odds ratio; MMP-9, matrix metalloproteinase 9; H6, hour 6; IV, intravenous; TICI, thrombolysis in cerebral infarction score; ICA, internal carotid artery*.

1 *per 1 mmol/L increase*.

2 *per 1°C increase*.

3 *per 30 min increase*.

*Significant values are shown in bold*.

### MMP-9 and Hemorrhagic Transformation

A high MMP-9 level at H6 [OR = 2.48 (1.16–5.27); *p* = 0.02] was associated with hemorrhagic transformation. A high WBC count [OR = 1.96 (0.91–4.25); *p* = 0.09] and a high hsCRP level [OR = 0.57 (0.28–1.27); *p* = 0.17] at admission were not associated with hemorrhagic transformation. After adjustment for main confounding factors, a high MMP-9 level at H6 [2.91 (1.14–7.42); *p* = 0.03], a high glucose level [1.20 (1.02–1.42); *p* = 0.03] and a long delay from symptom onset to groin puncture [OR = 1.08 (1.02–1.14) per 30 min increase; *p* = 0.02] remained significantly associated with hemorrhagic transformation.

## Discussion

Our study assessed the association between MMP-9 level and outcome in AIS patients with LVO treated with MT. The study design stands apart from earlier works due to a sequential assessment of both MMP-9 and ischemic damage on MRI. MMP-9 level at 6 h from admission was associated with infarct growth and hemorrhagic transformation.

We observed an early peak at 6 h from admission. Previous studies assessing kinetics of MMP-9 in AIS patients whether or not they are treated with IV thrombolysis with heterogeneous delays from the stroke onset have shown an early increase in the first 24 h (4, 10, 11, 18–22).

Pathological data show the presence of high MMP-9 levels not only in infarct tissue but also in the peri-infarct areas, suggesting MMP-9 involvement in the process of infarct growth (23, 24). A previous study conducted in 24 patients with middle cerebral artery (MCA) occlusion treated with IV thrombolysis found that MMP-9 level was associated with infarct growth at 24 h, but they did not report reperfusion status, which is a major confounding factor when interpreting their results (12). Our study confirms that MMP-9 level at H6 and infarct growth remains associated in the setting of MT after adjustment for reperfusion status.

Numerous studies have documented an increase in MMP-9 levels following AIS, associated with disruption of the BBB, thus promotion of hemorrhagic complications (2, 3, 25). This aspect has received special attention in patients treated with IV thrombolysis (4–9). Indeed, in addition to its thrombolytic action, tissue plasminogen activator (tPA), via activation of MMP-9, may also damage the basal lamina and tight junctions of the cerebral blood vessels, resulting in increased permeability of the BBB and hemorrhagic complications (26). We add to these existing data of MMP-9 activity and hemorrhage risk in AIS patients treated with tPA by examining AIS patients with LVO treated with MT, a therapy with much higher reperfusion rates and one which allows the recording of reperfusion status after treatment. We found an association between MMP-9 level at H6 and the risk of hemorrhagic transformation, mainly minor. The clinical relevance of this minor bleeding is still debated (27).

The observed association between MMP-9 level at H6 and infarct growth and hemorrhagic transformation do not necessarily imply a cause-effect relationship. Nevertheless, the experimental data currently available on the role of MMP-9 and on the effect of MMP-9 inhibition may be consistent with a causal relationship (28–32). Preclinical animal studies suggest that MMP-9 inhibition can be of therapeutic importance in ischemic stroke although a small pilot study conducted in humans did not show efficacy of this drug on 3-months mRS score in the setting of IV thrombolysis (28–33). Insofar as we have now entered into a new era of highly effective reperfusion, a new approach investigating the potential benefit of compounds which can directly inhibit MMP-9 activity should be considered in future MT trials (34).

We recognize some limitations of our study. First, although the limited sample size and the monocentric design may be considered as a limitation, its major strength lies in sequential assessment of MMP-9 coupled with MRI data within a homogeneous cohort of stroke patients with LVO in the context of MT. Secondly, imaging was performed either on 1.5 or 3 T according to MRI magnets availability. However, overall differences in the DWI and FLAIR imaging are usually subtle between both fields strengths as previously documented (35–37). Thirdly, final FLAIR-volume on day 6 may include a significant amount of edema instead of true infarction although previous studies have reported that it likely reflects final infarct size (38–40). The edema component could be further assessed using non-linear co-registration methods (41). Edema component should be further assessed using post-processing analysis as the nonlinear registration method (41). Fourthly, susceptibility-weighted imaging (SWI) or T2^*^-weighted gradient echo (GRE) imaging were not performed at day 6. These sequences would have been more sensitive than CT, and might have revealed minor hemorrhagic transformation (Hemorrhage infarction type 1 and 2) consistent with delayed reperfusion damage following blood brain barrier injury. Lastly, a comprehensive imaging assessment of BBB disruption assessing subarachnoid hemorrhage, gadolinium sulcal enhancement [hyperacute injury marker (HARM)], or microvascular permeability (K2) would have been a more direct measure of MMP-9 action and deserves further investigation (42–44).

In this MT cohort study using sequential assessment of MMP-9 levels and MRI, a high MMP-9 level at H6 predicts infarct growth and hemorrhagic transformation.

## Data Availability Statement

All datasets generated for this study are included in the article/supplementary material.

## Ethics Statement

The studies involving human participants were reviewed and approved by the study was approved by the local ethics committee and all subjects or their relatives signed an informed consent form. The patients/participants provided their written informed consent to participate in this study.

## Author Contributions

LM and TB: major role in the acquisition of data, analysis of the data, drafting the manuscript for intellectual content, AP, CC, MBo, LD, EO, YB, OE, and ND: major role in the acquisition of data, revised the manuscript for intellectual content, MBu: major role in the acquisition of data, analysis of the data, revised the manuscript for intellectual content, CA: analysis of the data, revised the manuscript for intellectual content, NM, MO: design of the study, revised the manuscript for intellectual content, TC: major role in the acquisition of data, analysis of the data, revised the manuscript for intellectual content, NN: design of the study, major role in the acquisition of data, drafting the manuscript for intellectual content.

## Conflict of Interest

The authors declare that the research was conducted in the absence of any commercial or financial relationships that could be construed as a potential conflict of interest.

## References

[1] Bai J Lyden PD. Revisiting cerebral postischemic reperfusion injury: new insights in understanding reperfusion failure, hemorrhage, and edema. Int J Stroke. (2015) 10:143–52. 10.1111/ijs.12434 25598025

[2] Pfefferkorn T Rosenberg GA. Closure of the blood-brain barrier by matrix metalloproteinase inhibition reduces rtPA-mediated mortality in cerebral ischemia with delayed reperfusion. Stroke. (2003) 34:2025–30. 10.1161/01.STR.0000083051.93319.28 12855824

[3] Barr TL Latour LL Lee K-Y Schaewe TJ Luby M Chang GS. Blood-brain barrier disruption in humans is independently associated with increased matrix metalloproteinase-9. Stroke. (2010) 41:e123–8. 10.1161/STROKEAHA.109.570515 20035078 PMC2827673

[4] Montaner J Molina CA Monasterio J Abilleira S Arenillas JF Ribó M. Matrix metalloproteinase-9 pretreatment level predicts intracranial hemorrhagic complications after thrombolysis in human stroke. Circulation. (2003) 107:598–603. 10.1161/01.CIR.0000046451.38849.90 12566373

[5] Castellanos M Leira R Serena J Pumar JM Lizasoain I Castillo J. Plasma metalloproteinase-9 concentration predicts hemorrhagic transformation in acute ischemic stroke. Stroke. (2003) 34:40–6. 10.1161/01.STR.0000046764.57344.31 12511748

[6] Castellanos M Sobrino T Millán M García M Arenillas J Nombela F. Serum cellular fibronectin and matrix metalloproteinase-9 as screening biomarkers for the prediction of parenchymal hematoma after thrombolytic therapy in acute ischemic stroke: a multicenter confirmatory study. Stroke. (2007) 38:1855–9. 10.1161/STROKEAHA.106.481556 17478737

[7] Rodríguez JA Sobrino T Orbe J Purroy A Martínez-Vila E Castillo J. proMetalloproteinase-10 is associated with brain damage and clinical outcome in acute ischemic stroke. J Thromb Haemost. (2013) 11:1464–73. 10.1111/jth.12312 23742289

[8] Inzitari D Giusti B Nencini P Gori AM Nesi M Palumbo V. MMP9 variation after thrombolysis is associated with hemorrhagic transformation of lesion and death. Stroke. (2013) 44:2901–3. 10.1161/STROKEAHA.113.002274 23908067

[9] Wang L Wei C Deng L Wang Z Song M Xiong Y. The accuracy of serum matrix metalloproteinase-9 for predicting hemorrhagic transformation after acute ischemic stroke: a systematic review and meta-analysis. J Stroke Cerebrovasc Dis. (2018) 27:1653–65. 10.1016/j.jstrokecerebrovasdis.2018.01.023 29598905

[10] Ning M Furie KL Koroshetz WJ Lee H Barron M Lederer M. Association between tPA therapy and raised early matrix metalloproteinase-9 in acute stroke. Neurology. (2006) 66:1550–5. 10.1212/01.wnl.0000216133.98416.b4 16717217

[11] Kelly PJ Morrow JD Ning M Koroshetz W Lo EH Terry E. Oxidative stress and matrix metalloproteinase-9 in acute ischemic stroke: the biomarker evaluation for antioxidant therapies in stroke (BEAT-stroke) study. Stroke. (2008) 39:100–4. 10.1161/STROKEAHA.107.488189 18063832

[12] Rosell A Alvarez-Sabín J Arenillas JF Rovira A Delgado P Fernández-Cadenas I A matrix metalloproteinase protein array reveals a strong relation between MMP-9 and MMP-13 with diffusion-weighted image lesion increase in human stroke. Stroke. (2005) 36:1415–20. 10.1161/01.STR.0000170641.01047.cc 15947272

[13] Mizuma A You JS Yenari MA. Targeting reperfusion injury in the age of mechanical thrombectomy. Stroke. (2018) 49:1796–802. 10.1161/STROKEAHA.117.017286 29760275 PMC6019565

[14] Adams HP Jr Bendixen BH Biller J Love BB Gordon DL Marsh EE. Classification of subtype of acute ischemic stroke. Stroke. (1993) 24:35–41. 10.1161/01.str.24.1.35 7678184

[15] Hacke W Kaste M Toni D Lesaffre E von Kummer R. Intravenous thrombolysis with recombinant tissue plasminogen activator for acute hemispheric stroke. JAMA. (1995) 274:1017–25. 10.1001/jama.1995.03530130023023 7563451

[16] Barber PA Demchuk AM Zhang J Buchan AM. Validity and reliability of a quantitative computed tomography score in predicting outcome of hyperacute stroke before thrombolytic therapy. Lancet. (2000) 355:1670–4. 10.1016/S0140-6736(00)02237-6 10905241

[17] Higashida RT Furlan AJ. Trial design and reporting standards for intra-arterial cerebral thrombolysis for acute ischemic stroke. Stroke. (2003) 34: e109–e37. 10.1161/01.STR.0000082721.62796.09 12869717

[18] Worthmann H Tryc AB Goldbecker A Ma YT Tountopoulou A Hahn A. The temporal profile of inflammatory markers and mediators in blood after acute ischemic stroke differs depending on stroke outcome. Cerebrovasc Dis. (2010) 30:85–92. 10.1159/000314624 20484906

[19] Demir R Ulvi H Özel L Özdemir G Güzelcik M Aygül R. Relationship between plasma metalloproteinase-9 levels and volume and severity of infarct in patients with acute ischemic stroke. Acta Neurol Belg. (2012) 112:351–6. 10.1007/s13760-012-0067-4 22581515

[20] Sobrino T Pérez-Mato M Brea D Rodríguez-Yáñez M Blanco M Castillo J. Temporal profile of molecular signatures associated with circulating endothelial progenitor cells in human ischemic stroke. J Neurosci Res. (2012) 90:1788–93. 10.1002/jnr.23068 22513751

[21] Brouns R Wauters A De Surgeloose D Mariën P De Deyn PP. Biochemical markers for blood-brain barrier dysfunction in acute ischemic stroke correlate with evolution and outcome. Eur Neurol. (2011) 65:23–31. 10.1159/000321965 21135557

[22] Montaner J Rovira A Molina CA Arenillas JF Ribó M Chacón P. Plasmatic level of neuroinflammatory markers predict the extent of diffusion-weighted image lesions in hyperacute stroke. J Cereb Blood Flow Metab. (2003) 23:1403–7. 10.1097/01.WCB.0000100044.07481.97 14663335

[23] Rosell A Ortega-Aznar A Alvarez-Sabín J Fernández-Cadenas I Ribó M Molina CA. Increased brain expression of matrix metalloproteinase-9 after ischemic and hemorrhagic human stroke. Stroke. (2006) 37:1399–406. 10.1161/01.STR.0000223001.06264.af 16690896

[24] Amantea D Russo R Gliozzi M Fratto V Berliocchi L Bagetta G Bernardi G Corasaniti MT. Early upregulation of matrix metalloproteinases following reperfusion triggers neuroinflammatory mediators in brain ischemia in rat. Int Rev Neurobiol. (2007) 82:149–69. 10.1016/S0074-7742(07)82008-3 17678960

[25] Rosenberg GA Yang Y. Vasogenic edema due to tight junction disruption by matrix metalloproteinases in cerebral ischemia. Neurosurg Focus. (2007) 22:1–9. 10.3171/foc.2007.22.5.5 17613235

[26] Sumii T Lo EH. Involvement of matrix metalloproteinase in thrombolysis-associated hemorrhagic transformation after embolic focal ischemia in rats. Stroke. (2002) 33:831–6. 10.1161/hs0302.104542 11872911

[27] Kaesmacher J Kaesmacher M Maegerlein C Zimmer C Gersing AS Wunderlich S. Hemorrhagic transformations after thrombectomy: risk factors and clinical relevance. Cerebrovasc Dis Basel Switz. (2017) 43:294–304. 10.1159/000460265 28343220

[28] Jiang X-F Namura S Nagata I. Matrix metalloproteinase inhibitor KB-R7785 attenuates brain damage resulting from permanent focal cerebral ischemia in mice. Neurosci Lett. (2001) 305:41–4. 10.1016/S0304-3940(01)01800-6 11356303

[29] Nagel S Su Y Horstmann S Heiland S Gardner H Koziol J. Minocycline and hypothermia for reperfusion injury after focal cerebral ischemia in the rat-effects on BBB breakdown and MMP expression in the acute and subacute phase. Brain Res. (2008) 1188:198–206. 10.1016/j.brainres.2007.10.052 18031717

[30] Switzer JA Hess DC Ergul A Waller JL Machado LS Portik-Dobos V Matrix metalloproteinase-9 in an exploratory trial of intravenous minocycline for acute ischemic stroke. Stroke. (2011) 42:2633–5. 10.1161/STROKEAHA.111.618215 21737808 PMC3181080

[31] Fan F Yang J Xu Y Guan S. MiR-539 Targets MMP-9 to regulate the permeability of blood-brain barrier in ischemia/reperfusion injury of brain. Neurochem Res. (2018) 43:2260–7. 10.1007/s11064-018-2646-0 30276507

[32] Romanic AM White RF Arleth AJ Ohlstein EH Barone FC. Matrix metalloproteinase expression increases after cerebral focal ischemia in rats: inhibition of matrix metalloproteinase-9 reduces infarct size. Stroke. (1998) 29:1020–30. 10.1161/01.STR.29.5.1020 9596253

[33] Kohler E Prentice DA Bates TR Hankey GJ Claxton A van Heerden J. Intravenous minocycline in acute stroke: a randomized, controlled pilot study and meta-analysis. Stroke. (2013) 44:2493–9. 10.1161/STROKEAHA.113.000780 23868273

[34] Savitz SI Baron J-C Yenari MA Sanossian N Fisher M. Reconsidering neuroprotection in the reperfusion era. Stroke. (2017) 48:3413–9. 10.1161/STROKEAHA.117.017283 29146878

[35] Rosso C Drier A Lacroix D Mutlu G Pires C Lehericy S. Diffusion-weighted MRI in acute stroke within the first 6 hours: 1.5 or 3.0 Tesla? Neurology. (2010) 74:1946–53. 10.1212/WNL.0b013e3181e396d1 20463287

[36] Kosior RK Wright CJ Kosior JC Kenney C Scott JN Frayne R Hill MD. 3-Tesla versus 1.5-Tesla magnetic resonance diffusion and perfusion imaging in hyperacute ischemic stroke. Cerebrovasc Dis. (2007) 24:361–8. 10.1159/000106983 17690549

[37] Kuhl CK Textor J Gieseke J von Falkenhausen M Gernert S Urbach H. Acute and subacute ischemic stroke at high-field-strength (3.0-T) diffusion-weighted MR imaging: intraindividual comparative study. Radiology. (2005) 234:509–16. 10.1148/radiol.2342031323 15601894

[38] Krongold M Almekhlafi MA Demchuk AM Coutts SB Frayne R Eilaghi A. Final infarct volume estimation on 1-week follow-up MR imaging is feasible and is dependent on recanalization status. NeuroImage Clin. (2015) 7:1–6. 10.1016/j.nicl.2014.10.010 25429356 PMC4238048

[39] Tourdias T Renou P Sibon I Asselineau J Bracoud L Dumoulin M. Final cerebral infarct volume is predictable by MR imaging at 1 week. Am J Neuroradiol. (2011) 32:352–8. 10.3174/ajnr.A2271 20966063 PMC7965712

[40] Lu M Mitsias PD Ewing JR Soltanian-Zadeh H Bagher-Ebadian H Zhao Q. Predicting final infarct size using acute and subacute multiparametric MRI measurements in patients with ischemic stroke. J Magn Reson Imaging. (2005) 21:495–502. 10.1002/jmri.20313 15834917

[41] Harston GWJ Carone D Sheerin F Jenkinson M Kennedy J. Quantifying infarct growth and secondary injury volumes: comparing multimodal image registration measures. Stroke. (2018) 49:1647–55. 10.1161/STROKEAHA.118.020788 29895538 PMC6023577

[42] Luby M Hsia AW Nadareishvili Z Cullison K Pednekar N Adil MM. Frequency of Blood-brain barrier disruption post-endovascular therapy and multiple thrombectomy passes in acute ischemic stroke patients. Stroke. (2019) 50:2241–4. 10.1161/STROKEAHA.119.025914 31238832 PMC6646098

[43] Renú A Laredo C Lopez-Rueda A Llull L Tudela R San-Roman L. Vessel wall enhancement and blood-cerebrospinal fluid barrier disruption after mechanical thrombectomy in acute ischemic stroke. Stroke. (2017) 48:651–7. 10.1161/STROKEAHA.116.015648 28174330

[44] Latour LL Kang D-W Ezzeddine MA Chalela JA Warach S. Early blood-brain barrier disruption in human focal brain ischemia. Ann Neurol. (2004) 56:468–77. 10.1002/ana.20199 15389899

